# Modulation of the Circulating Extracellular Vesicles in Response to Different Exercise Regimens and Study of Their Inflammatory Effects

**DOI:** 10.3390/ijms24033039

**Published:** 2023-02-03

**Authors:** Serena Maggio, Barbara Canonico, Paola Ceccaroli, Emanuela Polidori, Andrea Cioccoloni, Luca Giacomelli, Carlo Ferri Marini, Giosuè Annibalini, Marco Gervasi, Piero Benelli, Francesco Fabbri, Laura Del Coco, Francesco Paolo Fanizzi, Anna Maria Giudetti, Francesco Lucertini, Michele Guescini

**Affiliations:** 1 Department of Biomolecular Sciences, University of Urbino Carlo Bo, 61029 Urbino, Italy; 2 Biosciences Laboratory, IRCCS Istituto Romagnolo per lo Studio dei Tumori (IRST) “Dino Amadori”, 47014 Meldola, Italy; 3 Dipartimento di Scienze e Tecnologie Biologiche ed Ambientali, Centro Ecotekne, Monteroni, 73047 Lecce, Italy

**Keywords:** extracellular vesicles, aerobic exercise, aerobic training, exerkines, myokines, miRNA expression

## Abstract

Exercise-released extracellular vesicles (EVs) are emerging as a novel class of exerkines that promotes systemic beneficial effects. However, slight differences in the applied exercise protocols in terms of mode, intensity and duration, as well as the need for standardized protocols for EV isolation, make the comparison of the studies in the literature extremely difficult. This work aims to investigate the EV amount and EV-associated miRNAs released in circulation in response to different physical exercise regimens. Healthy individuals were subjected to different exercise protocols: acute aerobic exercise (AAE) and training (AT), acute maximal aerobic exercise (AMAE) and altitude aerobic training (AAT). We found a tendency for total EVs to increase in the sedentary condition compared to trained participants following AAE. Moreover, the cytofluorimetric analysis showed an increase in CD81^+^/SGCA^+^/CD45^−^ EVs in response to AAE. Although a single bout of moderate/maximal exercise did not impact the total EV number, EV-miRNA levels were affected as a result. In detail, EV-associated miR-206, miR-133b and miR-146a were upregulated following AAE, and this trend appeared intensity-dependent. Finally, THP-1 macrophage treatment with exercise-derived EVs induced an increase of the mRNAs encoding for *IL-1β*, *IL-6* and *CD163* using baseline and immediately post-exercise EVs. Still, 1 h post-exercise EVs failed to stimulate a pro-inflammatory program. In conclusion, the reported data provide a better understanding of the release of circulating EVs and their role as mediators of the inflammatory processes associated with exercise.

## 1. Introduction

Regular physical exercise promotes systemic adaptations that positively affect the cardiovascular [1], nervous [2] and immune systems [3], promotes weight loss [4] and counteracts sarcopenia [5]. Moreover, the level of physical activity is the major modifiable risk factor for metabolic [6] and cancerous diseases [7].

Skeletal muscle has recently been identified as an endocrine organ that produces and releases factors with paracrine, autocrine or endocrine effects. One mechanism by which physical activity induces systemic benefit is the release of cytokines from the contracting skeletal muscle termed myokines [8,9]. Using computational and proteomic approaches, more than 300 putative secretory proteins have been identified as putative myokines released by human skeletal muscle during exercise [10,11].

Until recently, exercise-induced benefits have been linked to the secretion of soluble proteins, however very recent studies have suggested that in addition to “classical” myokines, contracting muscle releases into the bloodstream other molecule types such as RNAs, miRNAs and mtDNA, which could be partly responsible for the benefits of physical activity on health. Safdar et al. [12,13] suggested the term “exerkines” for the total of all factors (including nucleic acids, peptides and metabolites) released in response to exercise stimulus.

In addition to soluble factors, extracellular vesicles (EVs) have emerged as a potential tool through which the muscle communicates with other tissues or organs [11,12,14,15,16,17].

Cells constitutively secrete EVs, but also after different extracellular signals like ATP, depolarization, lipopolysaccharide and neurotransmitters [15] controlled by Ca^2+^ signaling. In addition, our group demonstrated that muscle cells release EVs both in vitro [18] and in vivo [17]. This evidence was further investigated by Rome et al. [19]. Furthermore, Frϋhbeis et al. [15] showed that exercise triggers a rapid release of EVs with the characteristic size of exosomes into the circulation and the release initiated in the aerobic phase of exercise. Recently Whitham et al. [11] provided evidence for the involvement of EV trafficking in inter-tissue communication during exercise through the release into the circulation of several candidate myokines contained in EVs.

Our group found that 5% of circulating EVs were positive for α-sarcoglycan, a muscle-enriched protein, and contained skeletal muscle-specific microRNAs (MyomiRNAs), suggesting that skeletal muscle release EVs in circulation [17]. Furthermore, Whitman and colleagues [11] showed that a 1-hr bout of cycling exercise in healthy humans induced a significant increase in circulating EVs that were taken up by the liver. Additionally, in a study by Annibalini and co-workers, it is reported that systemin EVs’ concentration was higher 2 h following a single bout of a flywheel-based iso-inertial resistance training session [20]. On the contrary, Lovett et al. [21], did not observe a significant change in EV size or number after two consecutive bouts of muscle-damaging exercise; similarly, the levels of EV markers remained unchanged after 8-week training in the elderly [22].

EVs are a heterogeneous group of lipid-encapsulated vesicles that play a key role in cell-to-cell communication. The potential of EVs is mainly due to their cargo. This “vesicular package” offers the protection of signals from degradation and allows the simultaneous delivery of several messages over distance. Moreover, since EVs’ content may reflect the origin cell condition, they are considered plausible biomarkers for a variety of diseases [23,24].

The EV cargo includes peptides, proteins, lipids, messenger RNAs (mRNA), microRNAs (miRNA) and DNA which is also altered following the exercise. Among biomolecules transferred by EVs, microRNAs have aroused more interest in the scientific community. MiRNAs are evolutionarily conserved endogenous RNAs (around 19–24 nucleotides) considered post-transcriptional regulators of gene expression.

They mainly act by binding complementary sequences of mRNA in recipient cells interfering with the translational mechanism, preventing or altering protein production [25].

Several miRNAs are expressed or enriched in a tissue-specific manner; for example, miRNAs specifically expressed in muscle tissue are conventionally called Myo-miRNAs. The following belong to this group: miR-1-3p, -133a-3p, -133b, -206, -208a-3p, -208b-3p, and -499a-5p, with miR-208a-3p being cardiac muscle-specific while miR-206 being skeletal muscle [26,27,28]. MyomiRNAs are key regulators of physiological muscle mechanisms, such as proliferation, differentiation, repair, regeneration, and remodeling [29,30]. In addition to MyomiRNAs, other non-muscle-specific miRNAs are involved in myogenic functions such as miR-486, miR-146, miR-29 and miR-23 [31,32,33].

Interestingly, miRNAs are differentially expressed following physical activity. For example, EV-miR-133 is upregulated following a single bout of moderate aerobic exercise [17] or after high-intensity interval exercise [34]. On the contrary, Silver et al., did not find a significantly altered expression of miR-1, miR-16, miR-23b and miR-133a/b in EVs after an acute bout of endurance exercise [35].

The reported evidence shows that many factors, such as exercise intensity and duration as well as participant fitness conditions, may influence the release of EVs and their cargo.

The aim of this paper was to investigate the modulation of human muscle-related miRNAs carried by EVs in response to different protocols of physical exercise in healthy sedentary human subjects: three-month aerobic training, maximum aerobic exercise and two-week high-altitude aerobic training. Our results suggest that EV levels and EV-miRNA cargo are differently modulated in response to the proposed exercise. In addition, our group is the first to study the effect of exercise-EVs on the immune system, in particular macrophages.

Thus, our study extends the knowledge on how exercise-induced adaptations may be communicated throughout the body, making EVs possible valuable biomarkers to study the molecular response to physical activity and providing valuable information for clinical practice.

## 2. Results

### 2.1. Acute Aerobic Exercise (AAE) and Aerobic Training (AT)

**Figure 1:**
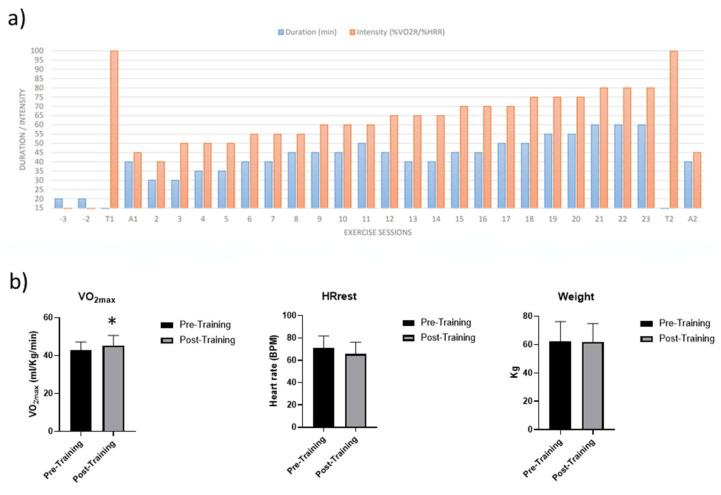
(**a**) Schematic representation of AT protocol. (**b**) VO_2max_ values, Heart rate at rest (HRrest) and body weight variations following aerobic training. Values are mean ± SD. * significant difference Pre- vs. Post-Training (*p* < 0.05, paired *t*-test).

Participants enrolled for the aerobic exercise protocol performed two months of moderate–intensity exercise 3 days/week. Each session consisted of about 40 min of aerobic exercise at 55% of their VO_2max_ (Figure 1a).

To monitor cardiorespiratory adaptations to the AT, VO_2max_ values were measured at sedentary and trained conditions. As reported in Figure 1b, VO_2max_ levels increased by 3–5% after two months, confirming the effectiveness of the AT protocol to improve aerobic fitness, while neither heart rate at rest (HRrest) nor body weight changed following AT (Figure 1b).

**Figure 2:**
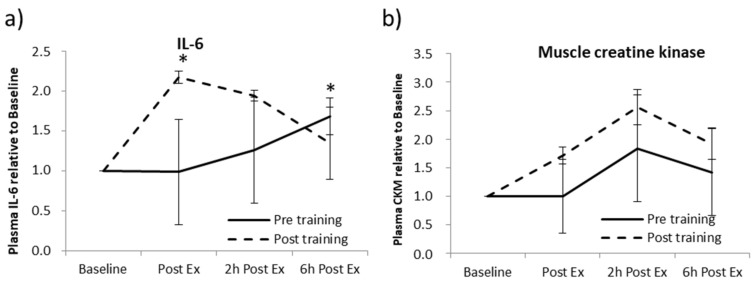
Modulation of IL-6 (**a**) and Creatine Kinase M-type systemic level (**b**) in response to an acute aerobic exercise in sedentary and trained subjects. The assays were performed at steady state (baseline), immediately after physical activity (Post Ex), after 2 (2 h Post Ex), and 6 h (6 h Post Ex) subsequently the end of the exercise. Values are mean ± SE. * significant difference from baseline (*p* < 0.05).

First, systemic levels of IL-6 were analyzed, as reported in Figure 2a. IL-6 levels showed an increase in both sedentary and trained conditions although the kinetics were different: IL-6 levels progressively increased during the exercise in the sedentary condition, whereas in the trained condition, IL-6 concentration was high immediately after exercise and then slowly decreased. The evidenced IL-6 upregulation suggests that a single exercise bout was sufficient to induce muscle secretory activity. On the contrary, circulating CMK levels showed a similar trend both in pre- and post-training conditions, showing a tendency to increase after 2 h from the end of the exercise bout (Figure 2b). 

**Figure 3:**
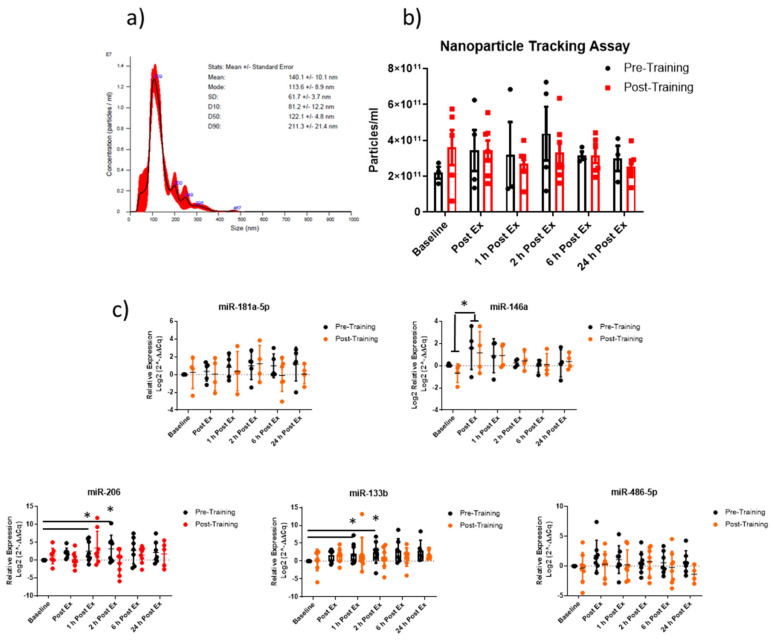
Acute aerobic exercise affects plasma EV concentration and EV-derived miRNAs. Typical size distribution plot of EVs isolated from plasma by serial ultracentrifugation (**a**). Quantification of the circulating EVs by NTA in sedentary and trained subjects at steady state (baseline), immediately after physical activity (Post Ex), after 1 h (1 h Post Ex), 2 h (2 h Post Ex), 6 h (6 h Post Ex), and 24 h (24 h Post Ex) subsequently the end of the exercise. The results are represented as mean ± SE (**b**). Expression profile of specific EV-miRNAs analyzed at baseline condition, immediately after physical activity and after 2, 6 and 24 h in pre- and post-training conditions. MiRNA expression levels were reported as log2(2^−ΔΔCq) (**c**). Values are mean ± SE. * significant difference from baseline (*p* < 0.05).

After having demonstrated that the administered exercise protocol induced the expected physiological adaptations in the studied volunteers, we focused on the study of circulating vesicles. EVs isolated from plasma were analyzed by the Nanoparticle tracking assay (NTA, Figure 3a) and the number of circulating EVs showed no significant variations between pre- and post-training conditions at baseline (Figure 3b). However, it is interesting to notice a tendency of EVs to increase after the first exercise session (sedentary condition) in the time-lapse between the start of physical stimulation and 2 h of recovery, while a blunted EV response in the trained condition was found (Figure 3b).

EVs are loaded with miRNAs, therefore, the expression profile of specific miRNAs purified from circulating EVs has been evaluated. Figure 3c shows a significant increase of miR-133b, -206 and -146a, while a tendency to increase was found for, miR-486-5p and -181a-5p within the first hour from the end of the exercise in the sedentary condition. 

Regarding the miRNA modulation after the training program, real-time qPCR data showed that the proposed exercise bout failed to upregulate EV-miRNAs except for miR-146, which increased immediately post-exercise. Overall, miRNA expression data suggest that an acute mild-intensity aerobic exercise induces the secretion of EVs with their miRNA cargo; however, in our condition, this stimulus was not sufficiently intense to trigger a similar response after the training period.

### 2.2. Acute Maximal Aerobic Exercise (AMAE)

The data found in the AAE and AT have underlined that the exercise effects on the EV release are more evident after an acute exercise session rather than regular exercise. To test whether the intensity of the exercise could regulate the levels of EV-miRNAs, the volunteers underwent acute maximal aerobic exercise (AMAE).

**Figure 4:**
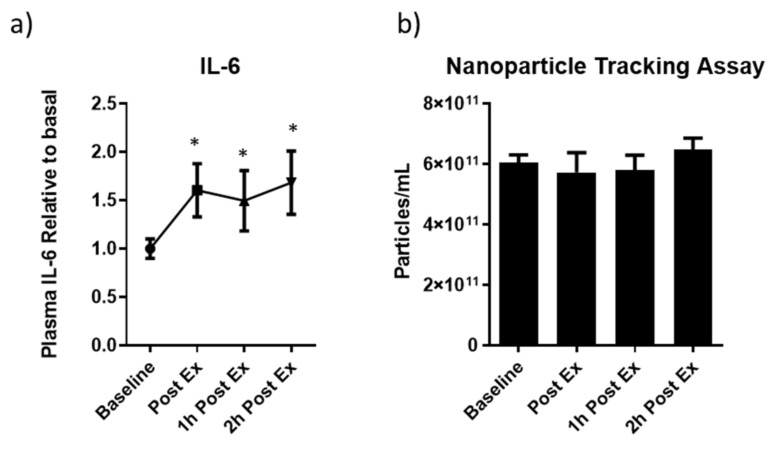
Evaluation of IL-6 systemic level in response to an acute maximal aerobic exercise (AMAE) in sedentary subjects at steady state (baseline), immediately after physical activity (Post Ex), after 1 (1 h Post Ex), and 2 h (2 h Post Ex) after the end of exercise (**a**). Quantification of the circulating EVs by NTA in response to AMAE in sedentary subjects at steady state (baseline), immediately after physical activity (Post Ex), after 1 h (1 h Post Ex), and 2 h (2 h Post Ex) subsequently the end of exercise (**b**). The results are represented as mean ± SE. * significant difference compared with baseline (*p* < 0.05).

The muscle adaptation by the proposed exercise was monitored by assessing circulating IL-6 levels. As expected, Figure 4a shows that the AMAE induced the increase of IL-6 systemic levels.

**Figure 5:**
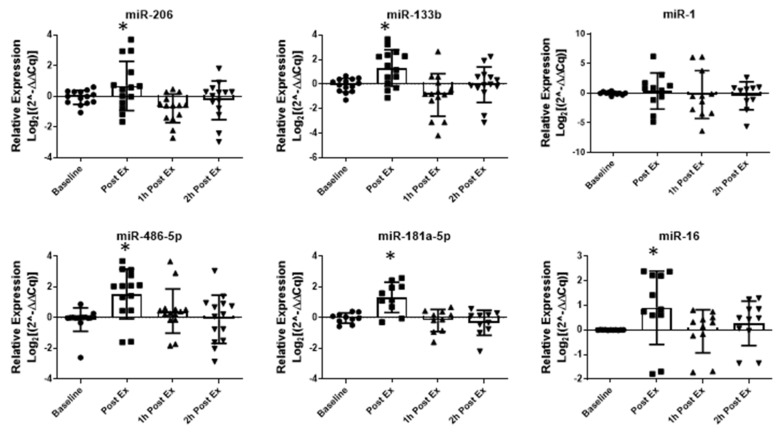
EV-miRNA quantification using real-time PCR following AMAE at baseline condition, immediately (Post Ex), after physical activity and after 1 (1 h Post Ex), and 2 h (2 h Post Ex) from the end of the exercise. MiRNA expression levels were reported as log2(2^−ΔΔCq). The results are represented as mean ± SE. * significant difference versus baseline (*p* < 0.05).

Although the NTA analysis of plasma EVs did not show changes in the particle number (Figure 4b) in response to AMAE, the study of the EV-miRNA expression displayed an increase immediately after exercise with a tendency to return to baseline levels faster if compared to the AAE. In detail, immediately after the acute exercise bout, three MyomiRNAs (miR-206, miR-133b and miR-486-5p) and two muscle-related miRNAs (miR-181a-5p and miR-16) were upregulated with the only exception being miR-1, which did not change during the time-window analyzed (Figure 5). In contrast to what we found after moderate aerobic exercise, the quick release of EV-miRNAs during an exhaustive incremental exercise may be related to the intensity of the exercise bout.

### 2.3. Altitude Aerobic Training (AAT)

**Figure 6:**
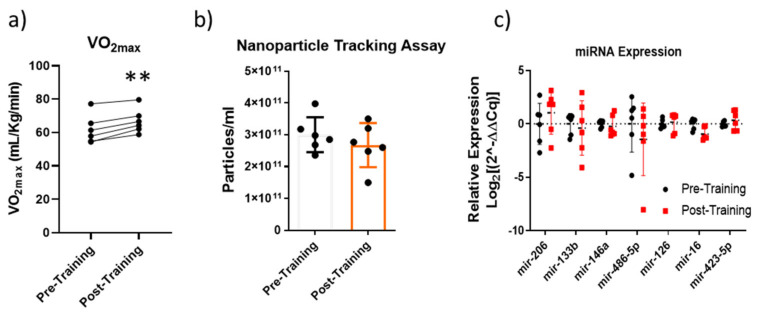
VO_2max_ values measured Pre- and Post-Training; ** significant difference Pre- vs. Post-Training *p* < 0.01, paired *t*-test (**a**). Nanoparticle tracking quantification of the circulating EVs pre- and post-AAT, the results are represented as mean ± SE (**b**). EV-miRNA quantification using real-time PCR following AAT; miRNA expression levels were reported as log2(2^−ΔΔCq) (**c**).

Six well-trained men participated in a 15-day training camp at 2000 m above sea level. After the training, aerobic capacity significantly increased (Figure 6a), whereas as reported in Figure 6b, the NTA demonstrated that AAT did not induce any increase in circulating EV number, which, conversely, seems to decrease. There were no statistically significant variations following the training regarding miRNA expression levels (Figure 6c).

The evidence that circulating EVs and EV-miRNAs remained unchanged following the AAT protocol could be due to the fact that volunteers were already well-trained at baseline, this condition could blunt the molecular response to the training.

### 2.4. Characterization of the Circulating EVs Released in Response to an Aerobic Acute Exercise

**Figure 7:**
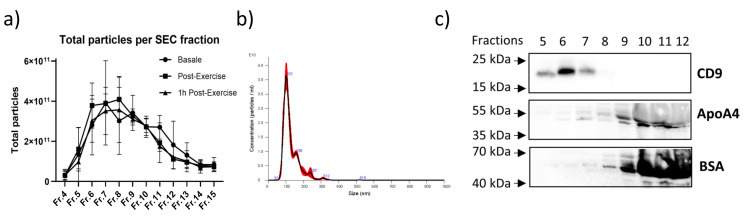
Size exclusion chromatography separation of EVs from plasma at baseline, Post-Exercise and 1 h Post-Exercise (**a**). NTA size distribution plot of EVs collected from Fr. 7 (**b**). Western blot analysis of Fr. 5–Fr. 12 using antibodies against CD9, ApoA4 and BSA (**c**).

The above-reported data suggest that EV-related signals accumulate in the bloodstream mainly immediately and up to 2 h after the end of aerobic exercise in sedentary conditions. With the aim to further characterize the molecular messages loaded into EVs and to shed light on the physiological effects triggered by exercise-induced EVs in mediating fitness adaptations, we isolated plasma-derived EVs using size-exclusion chromatography at baseline, post-exercise, and after 1 h from the end of the exercise bout. NTA confirmed the accumulation of particles in fractions 7, 8, and 9 (Figure 7a); moreover, the distribution plot of particle size confirmed the presence of tiny vesicles of about 120 nm in diameter (Figure 7b). Finally, western blot analysis demonstrated the presence of CD9-positive EVs in fractions 5, 6, and 7. At the same time, these fractions showed very low levels of contaminants such as albumin and APOA4, a lipoprotein marker (Figure 7c). Altogether these data demonstrate that we were able to isolate circulating EVs for further studies.

**Figure 8:**
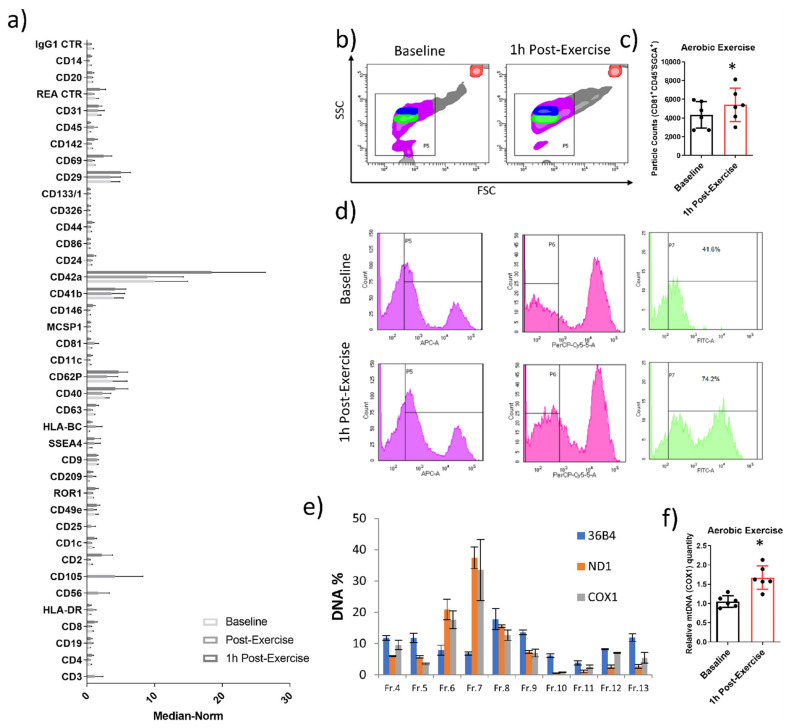
MACS plex analysis for each EV marker of circulating vesicles isolated by SEC (Fr. 7) at baseline and following aerobic exercise (immediately and 1 h after the end of exercise bout) (**a**). Contour plot FSC vs. SSC of EV preparations derived from baseline (left panel) and post-exercise (right panel) samples. Green and blue clusters represent the area of beads sized 500 nm and 1 micron, respectively. Such beads were acquired previously and dispersed in the filtered buffer in which each sample is prepared (see Supplementary Materials Figure S2). The red circle represents counting beads (DakoCyto-Count), whereas pink and violet events represent the EVs further characterised for CDs expression (**b**). Then, a protocol of gating strategy is applied in (**b**), both on the baseline and post-exercise samples: Violet events (histogram) are those from the area identified using size beads; Pink events (histogram) are those enclosed by P5, drawn in the violet histogram, virtually corresponding to appropriate-sized events, positive for CD81. This histogram focuses on CD45 expression. Green events (histogram) are those enclosed by P6, drawn in the pink histogram, virtually corresponding to appropriate-sized events, positive for CD81, and SGCA and negative for CD45 (**d**). The absolute counts of CD81^+^/CD45^−^/SGCA + EVs are reported in the subfigure (**c**). Real-time PCR quantification of mitochondrial (*ND1* and *COX1*) and nuclear (*36B4*) genes in SEC fractions (**e**) and in Fr. 7 obtained from plasma samples of baseline and 1 h post-exercise subjects (**f**). The results are represented as mean ± SE. * significant difference from baseline (*p* < 0.05).

Flow cytometry analysis of circulating EVs was then performed. On the whole, the EV marker expression showed an increase at 1 h post-exercise (Figure 8a). In particular, we found high expression levels of CD42a, CD41b, CD29, CD40 and CD62P; for these markers, the baseline and immediate post-exercise levels were high and further increased at 1 h after the end of exercise. Interestingly, CD56, CD105 and CD3 were detectable only immediately after exercise bout (Figure 8a). 

Muscle-derived EV secretion following exercise was studied to further characterize the crosstalk between muscle and other organs through EVs [17,36,37]. Based on the above reported NTA data showing that total circulating EVs mainly increase immediately up to 2 h after the end of first exercise bout, the release of muscle-derived vesicles after 1 h of recovery was quantified. Figure 8b shows the presence in the bloodstream of EVs simultaneously CD81^+^, SGCA^+^ and CD45^−^. Although the NTA analysis showed only a tendency for the total plasma EVs to increase in response to exercise bout (Figure 4b), our cytofluorimetric protocol allowed us to find an increment of the muscle-derived EVs (Figure 8b–d).

It has been recently reported that a single bout of running or cycling exercise increases the levels of EVs, cfDNA, and EV-associated DNA [38]. Here, we aimed to confirm and extend these data to the mitochondrial DNA, therefore we performed *ND1*, *COX1* (two mitochondrial genes) and *36B4* (a nuclear gene) quantification in the fractions of plasma obtained by SEC. Quantitive Real-time PCR data confirmed that the highest levels of mtDNA were found in fractions 6 and 7, demonstrating that the mtDNA is associated with EVs. Furthermore, the comparison between baseline and 1 h post-exercise levels of fraction 7 revealed an increase of EV-associated mtDNA in response to aerobic exercise (Figure 8e,f). On the whole, the characterization of the circulating EVs released following aerobic exercise shows that molecular signals carried by EVs are modulated by exercise stimulation and could have a role in mediating exercise-induced physiological adaptations.

### 2.5. Inflammatory Activity of Exercise-Derived EVs on THP-1 Cell Line

**Figure 9:**
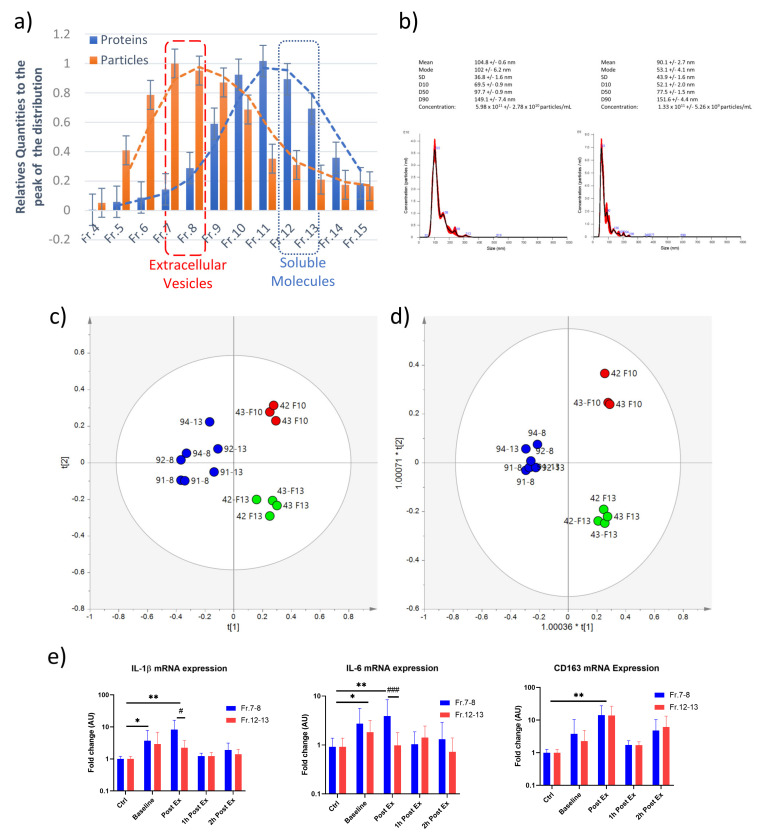
Size exclusion chromatography separation of EVs from plasma, separated EVs were quantified using NTA, whereas soluble factors were estimated by using Bradford protein assay (**a**). Size distribution plot of EVs collected from Fr. 7 and Fr. 13 (**b**). Partial least squares discriminant (PLS-DA) (**c**) and orthogonal partial least squares discriminant (OPLS-DA) (**d**) analyses performed on a partial dataset of EV lipid extracts. (**e**) Inflammatory effect of vesicular (Fr. 7–8) and soluble (Fr 12–13) factors isolated following acute aerobic exercise; THP-1 cells were incubated for 18 h with circulating factors and mRNA expression levels *IL-1β*, *IL-6* and *CD163* were evaluated by real-time qPCR. ANOVA test followed by Dunnett’s post hoc analysis vs. Ctrl; * p ≤ 0.05; ** p ≤ 0.001; *# p* ≤ 0.05, *### p* ≤ 0.001 Fr 7–8 vs. Fr 12–13).

Finally, we aimed to investigate the effects of the vesicular signals secreted following exercise on immune cells. At first, the EVs and soluble molecules released at baseline, immediately post-exercise, after 1 h and 2 h from the end of an aerobic exercise bout were separated by SEC. The quantity of EVs was evaluated using NTA, in contrast, the elution of soluble molecules was detected by quantifying total proteins (Figure 9a). The size distribution plots of nanoparticles from fractions 7–8 and 12–13 confirmed EV enrichment in the first eluted fractions (Figure 9b). In an attempt to further characterize vesicle- and soluble-associated molecules, lipid extracts from fractions 8, 10, and 13 were analyzed using NMR Spectroscopy (see Supplementary Materials, Figure S1 and Table S3). Then, an unsupervised multivariate statistical approach was applied to the NMR data to reveal the samples’ natural trend or data grouping. The unsupervised PCA analysis was performed to investigate the differences among samples, without a specific label, after the pre-processing treatment of the NMR spectra. The PCA model (Figure 9c and Supplementary Materials), obtained using the two first principal components (t [1]/t [2]), explains more than 70% of the total variance (R^2^X = 0.71, Q^2^ = 0.35). The unsupervised model allowed us to obtain a good separation, which became more evident by performing supervised PLS-DA and OPLS-DA analyses and considering a partial dataset (Figure 9c,d). The lipidomic characterization clearly identified three groups of samples suggesting a characteristic lipid composition for the vesicular and soluble fraction. Then to assess the inflammatory properties of the circulating factors, THP-1 cells were incubated for 18h with Fr. 7–8 (EVs) or Fr. 12–13 (soluble factors) at a concentration of 5 µg/mL. As shown in Figure 9e, both EVs and soluble factors released in the bloodstream at the baseline and immediately post-exercise induced a significant upregulation of the *IL-1β*, *IL-6* and *CD163* mRNA levels. On the contrary, when THP-1 cells were treated with 1 h and 2 h post-exercise factors, these mRNAs remained like control levels (untreated condition, Figure 9e). The mRNA expression levels of the studied markers showed a very similar trend for both Fr. 7–8 and Fr. 12–13, except for *IL-1β* and *IL-6* mRNA modulation in the immediately post-exercise condition, in which we highlighted a significant *IL-1β* and *IL-6* increase in the presence of vesicular (Fr 7–8) but not soluble (Fr. 12–13) factors suggesting that EVs showed higher pro-inflammatory activity than soluble molecules.

## 3. Discussion

Physical exercise is associated with immediate changes in different physiological parameters that are largely considered beneficial to health. A growing body of evidence demonstrates that exercise generates widespread systemic effects leading to protection from metabolic diseases in many organs beyond skeletal muscle. Several studies have described signaling pathway networks and regulatory molecules that coordinate these adaptive responses to exercise. In this view, the skeletal muscle acts as a secretory organ during exercise, producing and releasing myokines into circulation that can affect other organs [10,11,12,13,39].

In the last years, a new mechanism of secretion stimulated by physical exercise and mediated by Extracellular Vesicle (EV) release is emerging. EVs are spherical structures bound by a lipid bilayer, similar in composition to the originating cell membrane. Vesicles carry signals in their limiting membrane or interior lumen, mediating adaptive responses over large distances with widespread implications in physiology [40]. Within the “vesicular package”, EVs selectively incorporate and protect miRNAs capable of targeting mRNAs in recipient cells [41].

In this study, we investigated whether different physical activity protocols affect the amount of circulating EVs and the levels of carried miRNAs. Interestingly, increasing evidence shows that circulating miRNAs may distinguish between specific stress signals imposed by variations in the duration, modality, and type of exercise and training [42,43]. The reported results highlighted that although the training protocol was sufficient to produce the cardio-respiratory adaptation, as proved by the significant VO_2max_ raise, the circulating EV amount does not change at rest before and after three months of mild aerobic training (sedentary versus trained condition). However, after a single exercise session of moderate intensity, a slight increase in the total plasma EV number was found in sedentary subjects but not in the trained ones, within 2 h after the end of the aerobic exercise. MACSplex data suggest that circulating EVs released following aerobic exercise are mainly associated with platelets, leucocytes, T, and endothelial cells, likely due to shear stress caused by increased blood flow on the walls of blood vessels. It is noteworthy that CD56 is a transmembrane glycoprotein expressed on the surface of neurons, glia, and skeletal muscles. The appearance of CD56 immediately post-exercise led us to hypothesize the release of muscle-derived EVs in circulation following the proposed aerobic exercise.

Although the amount of muscle-derived EVs in circulation is very low, cytofluorimetric data and myo-miRNA quantifications showed a rise of muscle-related signals carried by EVs in the bloodstream after a single bout of exercise in sedentary subjects. Moreover, in response to exhaustive incremental exercise, the increase of muscle-related miRNAs was more evident and occurred faster compared to AAE, suggesting that the rise of systemic EV-miRNAs is exercise intensity dependent.

These data agree with the current literature on circulating miRNAs. For example, a single bout of high-intensity resistance exercise increased miR-133 and -206 [34]; miRNA-1, -133a, -133b, and -206 were also significantly increased during high-intensity interval exercise and vigorous-intensity continuous exercise [44]. Also, after a marathon and middle marathon, muscle-specific miRNAs increased immediately and 24 h post-exercise [45,46]. Moreover, a significant increase in EV-associated miRNA-206 and -146a was shown after the completion of a single bout of flywheel exercise [20]. On the contrary, Aoi et al. [47] reported no change in mir-1, -133a and -133b in healthy men undergoing cycling at 70% of VO_2max_ for 60 min.

Even though there are growing studies of circulating miRNAs in fitness, little is known about miRNAs packed in EVs released in response to exercise or training. This point deserves attention because there is clear evidence that vesicular miRNAs can be internalized by a target cell regulating its gene expression [41,48], while it is still unclear whether free miRNA acts similarly.

Among the studied miRNAs, miR-206 and -133b have been involved in promoting myogenic differentiation [49]. Furthermore, miR-206 modulates neuromuscular junction development [50] and regulates the regeneration of neuromuscular synapses [51]. Therefore, once taken up by the target cell, these miRNAs could mediate muscle plasticity in response to physical activity.

In the context of metabolic diseases, numerous studies have demonstrated the involvement of EVs in health (i.e., development and tissue homeostasis) and diseases (i.e., cancer metastasis, inflammatory diseases, and metabolic diseases). EVs are involved in inflammation, coagulation, vascular function, and sepsis [52,53]. Regarding muscle, it was found that myotubes, pre-treated with H_2_O_2_ mimicking oxidative stress, release EVs carrying pro-inflammatory signals that stimulate myoblast proliferation and decrease myotube size [54]. Similar results were obtained when myotubes were treated with a pro-inflammatory cytokine mixture of TNF-alpha and INF-gamma [29], suggesting that in addition to myogenesis, muscle-derived EVs would also participate in the maintenance and regeneration of muscle following injuries.

Herein, we demonstrated that circulating EVs are loaded with miR-146a, a miRNA involved in the regulation of multiple functions relevant to inflammation, cardiovascular diseases, and exercise [55], and mtDNA, which is associated with pro-inflammatory conditions when released outside the cell. Furthermore, it has been shown that miR-146a targets a variety of molecules belonging to the NF-κB/NLRP3 [56]. Interestingly, THP-1 macrophage treatment with circulating EVs revealed an increase in the *IL-1β*, *IL-6*, and *CD163* mRNA levels in baseline and immediately post-exercise conditions. Furthermore, when the same experiment was repeated treating THP-1 cells with vesicular (SEC Fr. 7–8) and soluble factors (SEC Fr. 12–13) separately, circulating EVs showed higher pro-inflammatory activity than soluble molecules. More interestingly, both circulating EVs and soluble factors released 1 h post-exercise failed to stimulate a pro-inflammatory program in the target cells, suggesting that molecules with anti-inflammatory activity, including EVs, could accumulate in circulation during the first hours of exercise recovery. Altogether, these data support the idea of exercise-induced EVs’ possible role in mediating inflammation processes, tissue regeneration, and homeostasis.

One of the limitations of this study is the level of AAE intensity set in the post-training condition; indeed, it appears that the administered stimulus failed to trigger EV-miRNA increase in the two-month trained subjects; another point refers to the AAT, neither circulating EV quantity nor EV-miRNA modulation changed after the AAT. These data could be due to the timing of the blood sampling; in fact, at 48 h from the last exercise stimulus, the clearance processes could hide the EV modulation. Further research will be needed to understand whether EV-associated pro-inflammatory signals are loaded into vesicles or recruited onto the EV surface during circulation. Other efforts will be directed towards identifying and characterizing the molecular signals associated with EVs responsible for the low inflammatory activity (possible anti-inflammatory activity) highlighted at 1 h post-exercise.

Nevertheless, the current study represents a step forward in comprehending the release of EVs in the bloodstream in response to AAE, AMAE, and AAT and their role in modulating exercise-associated inflammation processes.

## 4. Materials and Methods

### 4.1. Subjects

All experiments were performed according to the Helsinki Declaration of 1975 as revised in 2008 on ethical principles for medical research involving human subjects. The project was approved by the Scientific Committee of University of Urbino (Approval Number 28507). Before enrolment, the subjects gave their written informed consent to participate in the study. Subjects’ physical activity level was first assessed based on their answers to questions asked in a simple face-to-face interview and then confirmed using a validated questionnaire.

### 4.2. Study Population

Inclusion criteria were age, between 20 and 50 years; not physically active, according to the questionnaire results, except for the AAT study in which well-trained subjects were enrolled. Exclusion criteria were chronic or recent (≤2 weeks) treatment with drugs acting on skeletal muscle; recent (≤3 months) history of traumatic muscle injury and history of cardiovascular disease. Thirteen volunteers were enrolled for the AAE, AT, and AMAE, the characteristics of these subjects are summarized in the Supplementary Materials Table S1. Six well-trained men participated in the 15-day training camp at 2000 m above sea level (AAT); the characteristics of these subjects are summarized in the Supplementary Materials Table S2.

### 4.3. Acute Aerobic Exercise (AAE) and Aerobic Training (AT)

This single-group intervention trial employed a longitudinal design aimed to assess whether switching from the sedentary to the trained condition affects blood acute responses to a single bout of moderate intensity aerobic exercise. After enrolment, participants underwent 2 familiarization sessions to get used to walking and running on the treadmill ergometers used for testing and training purposes. Baseline anthropometric data were measured before the exercise bout of the second familiarization session. Participants were then scheduled for 2 testing sessions (T1 and A1) separated by at least 4 days. They were asked to avoid any physical activity and to maintain usual dietary habits in the 4 days before both T1 and A1. In T1, participants’ baseline body composition, pre-exercise heart rate (HR), maximal HR and maximum oxygen uptake (V̇O_2max_) were assessed, while A1 consisted of a steady-state aerobic exercise bout. Before and after the A1 blood samples were repeatedly collected (see below). Those 2 testing sessions were repeated (T2 and A2), using the same exact procedures, after participants completed a gradual, moderate-to-vigorous intensity exercise training intervention of 8 weeks. A schematic representation of the experimental design is shown in Figure 1a.

### 4.4. Acute Maximal Aerobic Exercise (AMAE)

The participants performed a graded exercise test (GXT) to exhaustion on a treadmill (Runrace model; Technogym, Cesena, FC, Italy) using a personalized incremental running protocol. The GXT started with a warm-up of 5 min by walking on the treadmill (set at 1% grade). During the fifth minute, the treadmill speed was gradually increased until the participant preferred to start running instead of walking. The preferred walk-to-run transition speed (PTS) was recorded and used as the initial speed of the GXT, which started immediately after the end of the warm-up. Treadmill grade was kept constant at 1% throughout the GXT, while the initial running speed was set at each participant’s PTS. The speed increment of each 1-min stage was calculated using a standard procedure (see Supplementary Materials), which aims to allow the attainment of the V̇O_2max_ approximately within 10 min from the beginning of the GXT. Strong verbal encouragement was provided throughout the GXT to push the participant to his/her own volitional maximal effort. For the duration of the test, V̇O_2_ was measured (breath-by-breath) using a metabolimeter (k4b2 model; Cosmed, Rome, Italy) and HR was constantly recorded. The highest smoothed values of V̇O_2_ and HR were assumed as V̇O_2max_ and HRmax, respectively.

### 4.5. Altitude Aerobic Training (AAT)

We enrolled six athletes, age 23.3 ± 6.8; weight 59.7 ± 10.4 kg; height 173.8 ± 12.2 cm; with training experience in the extended middle-distance event (5000–10,000 m) of 5 ± 1 years. Each athlete was tested to determine his VO_2max_, lactate threshold (LT1, LT2) and individual training zones. Training intensity zones for each athlete were based on the following lactate concentration values: zone 1 (<LT1), <2.0 mmol/L, zone 2 (>LT1 <LT2), >2.0 and <4.0 mmol/L, zone 3 (>LT2), >4.0 mmol/L, as proposed by Seiler and Kjerland [57]. The protocol to determine the V̇O_2max_ and LT1-2 consisted of a minimum of 5 and a maximum of 9 incremental 3 min stages. The intensity of the first step was calculated according to the individual athletic level, and the speed of the treadmill was increased by 1 km/h every 3 min until exhaustion, as indicated in the guidelines. Oxygen uptake was monitored for the duration of the trial (breath-by-breath) using the Cosmed k4b2 metabolimeter (COSMED, Rome, Italy). Lactate concentration was measured by micro-withdrawals from the fingertip of the forefinger with a Lactate Pro lactate meter (ARKRAY, Kyoto, Japan) [58] before the test and within the 30 s of the end of each stage. The running speed at LT1 (2.0 mmol/L) and LT2 (4.0 mmol/L) was determined using a validated algorithm by Bentley et al. [59] and implemented using specifically designed software by Newell et al. [60]. Athletes performed 23 sessions at a medium altitude (2000 m a.s.l.) of specific training in 15 days. To maximize potential endurance performance gains, the training program that athletes performed was designed following the guidelines reported by several studies [57,61] which describe the training method of the best middle-distance runners, defined as a polarized training model.

### 4.6. Blood Sampling

For AAE e AT blood samples were collected before the exercise session (baseline), immediately after physical activity (post-ex), and after 1, 2, 6, and 24 h, subsequently at the end of the exercise bout. Blood sampling was performed after the first acute aerobic exercise bout (AAE, sedentary condition) and following the last acute aerobic exercise bout performed after the three-month training period (AAE, trained condition).

For AMAE blood samples were collected one hour before the AMAE (baseline), immediately post-exercise, and after 1 and 2 h of recovery.

For AAT, blood samples were collected at sea level under baseline conditions, before leaving for the camp and at the end of the training program, at least at 48 h from the last session of exercise in order to exclude the acute effect of exercise.

Blood was drawn from the antecubital vein in EDTA tubes (12 mL Becton Dickinson, Franklin Lakes, NJ, USA) and centrifuged for 15 min at ~1000× *g* within 30 min of collection, at room temperature, then centrifuged again for 5 min at ~2000× *g* at 4 °C. Subsequently, aliquots were frozen at −80 °C.

### 4.7. IL-6 and CMK Activity ELISA Assays

The total plasma concentration of muscle creatine kinase (CK-M) and IL-6 at pre- and post-exercise; 2-, 6- and 24 h post-exercise was quantified using a commercially available ELISA kit (CK-M: SEA109Hu Cloud-Clone Corp, Houston, TX, USA; IL-6 HS600B R&D Systems, Minneapolis, MN, USA). Data were acquired at a wavelength of 450 nm using a microplate reader for a 96-well plate (Model 680, Bio-Rad Laboratories, Hercules, CA, USA). Samples derived from the same subject were quantified in the same assay.

### 4.8. Extracellular Vesicle Isolation

The EV isolation was carried out following the Minimal Information for Studies of Extracellular Vesicles guidelines developed by the International Society for Extracellular Vesicles (ISEV) in 2018 [62]. Plasma was cleared by centrifugation for 15 min at 1000× *g* to eliminate cell contaminations. Supernatants were further centrifuged for 30 min at 12,000× *g* and subsequently for 30 min at 18,000× *g*. The resulting supernatants were pelleted by ultracentrifugation at 110,000× *g* for 70 min. The EV pellet was washed in 10 mL PBS, centrifuged again, and resuspended in PBS. Size exclusion chromatography was performed using IZON (IZON, Lyon, France) column following the manufacturer’s instructions. For western blot analysis, samples containing 10–30 µg of protein were mixed with Laemmli sample buffer (1:1 ratio) and loaded onto 10% SDS-PAGE gels. Then, proteins were blotted to a Polyvinylidene difluoride (PVDF) membrane (Thermo Fisher Scientific, Milano, Italy). Primary antibodies used were: CD9 (1:1000 dilution, clone D801A, Cell Signalling Technology, Danvers, MA, USA), Albumin (1:1000 dilution, #4929, Cell Signalling Technology), and APOA4 (1:2000 dilution, clone ab59036, Abcam, Cambridge, UK). Primary antibodies were incubated overnight at 4 °C, followed by washing and the application of secondary HRP-conjugated antibody. Immune complexes were visualized using the Clarity and/or Clarity Max (Bio-Rad, Hercules, CA, USA).

### 4.9. Nanoparticle Tracking Assay (NTA)

NTA measurements were performed with a NanoSight LM10 (NanoSight, Amesbury, UK) and three videos of either 30 or 60 s were recorded of each sample. All measurements were performed at room temperature, never above 25 °C. The software used for capturing and analyzing the data was the NTA 3.1 (Nanosight). Data are presented as the mean ± SD of the three video recordings. Samples containing high particle numbers were diluted before analysis and the relative concentration was then calculated according to the dilution factor. Control 100 and 400 nm beads, supplied by Malvern Instruments Ltd. (Malvern, UK), were used.

### 4.10. Cytofluorimetric Analysis

For the Flow Cytometric characterization, EVs were purified from human plasma by SEC. The EVs were resuspended in PBS + 0.1% BSA and then stained with an anti-CD81 PE (BDPharmingen, clone JS-81) or anti-alpha-sarcoglycan (SGCA, clone AD1/20A6 Monosan) labelled with FITC and anti-CD45 (BDPharmingen) PE-Cy5. The cytometric analyses were performed by gating events smaller than 1 mm. Size beads (Ø 1–2 mm Polysciences Invitrogen, and Ø 5.2 mm DakoCyto-Count beads) were used to establish the proper gate for events smaller than 1 mm, which include EVs, and to obtain single platform absolute counts. A cytofluorimetric experimental protocol was developed to detect muscle-derived EVs (for more details, see also Supplementary Materials, Figure S2). Briefly, total plasma EVs were labeled with antibodies against CD81, SGCA and CD45. CD81 is a well-known EV marker and was used to reveal in particular exosomes [36], SGCA, a muscle-specific protein allowed us to specifically identify muscle-derived EVs, among total ones [17], whereas CD45, which is not expressed by muscle cells [37], help us to subtract not muscle-derived vesicles from the analysis. Thus, by quantifying the CD81^+^/SGCA^+^/CD45^−^ EVs from plasma, it was possible to estimate circulating EVs released from muscle tissue.

### 4.11. miRNA and DNA Quantifications

Total RNA from the EV pellet was extracted using the Zol RNA DIRECT CLEAN UP kit (FMB). The cDNA was synthesized using the Universal cDNA Synthesis Kit II (Exiqon). The q-PCRs were performed starting from cDNA with a miRCURY LNA Universal RT microRNA PCR system exiLENT SYBR Green (Exiqon) using miRCURY LNA uniRT PCR Primer (Exiqon) specific for human miRNAs analyzed. The real-time PCR conditions used were the following: 95 °C for 10 min followed by 50 cycles consisting of two steps at 95 °C for 10 s and 60 °C for 60 s. Cq were determined using the Cy0 method, performed according to the ΔCq method.

### 4.12. MACSPlex Analysis

The MACSPlex Exosome Kit (Miltenyi Biotec, Bergisch Gladbach, Germany) allows the detection of 37 exosomal surface epitopes. The MACSPlex Exosome Detection Reagents for CD9, CD81 and CD63 were used to label the captured exosomes. Briefly, 6 µL and 80 µL of EVs from serum were added to 114 µL and 40 µL of MACSPlex buffer, respectively, to obtain a final reaction volume of 120 µL. All samples were processed following the manufacturer’s instructions.

### 4.13. NMR Data Processing

The ^1^H NMR spectra were processed using Topspin 3.6.1 and Analysis of Mixture Amix 3.9.13 (Bruker, Biospin, Italy) software, both for simultaneous visual inspection and the statistical bucketing process. The entire NMR spectra (in the range 10.00–0.5 ppm) were segmented in fixed rectangular buckets of 0.04 ppm width and successively integrated. The spectral region between 7.44–6.28 and 1.80–1.48 ppm was discarded, excluding the residual peaks of chloroform and water, respectively. The total sum normalization was applied to minimize small differences due to sample concentration and/or experimental conditions among samples. Multivariate statistical analysis unsupervised principal component analysis (PCA), supervised partial least squares discriminant analysis (PLS-DA), and orthogonal partial least squares discriminant analysis (OPLS-DA) were performed to examine the intrinsic variation in the data, using SIMCA 14 software, (Sartorius Stedim Biotech, Umeå, Sweden) [63,64,65,66,67,68,69]. The Pareto scaling procedure was applied by dividing the mean-centered data by the square root of the standard deviation [4].

### 4.14. THP-1 Treatment and Gene Expression Analysis

THP-1 cells were cultured to a final density of 1 × 10^6^ cells/mL treatment condition. Cells were incubated with 5 µg of EVs or soluble proteins for 18 h. The untreated cells represented the negative controls. Total RNA from treated cells was extracted according to the RNAZOL DIRET CLEAN UP KIT manual (Fisher Molecular Biology, Rome, Italy). Reverse transcription of cDNA was performed from 200 ng of total RNA according to the PrimeScript™ RT Reagent Kit manual (Takara Bio Europe, Saint-Germain-en-Laye, France). From mRNA expression analyses, the cDNA products were subjected to Real-Time SYBR-green-based quantitative PCR on a Step One Plus™ Real Time PCR System (Applied Biosystems, Monza, MB, Italy). The list of the primers used for cDNA amplification: *36B4* as the reference gene (Forw: 5′-CGACCTGGAAGTCCAACTAC-3′, Rev: 5′-ATCTGCTGCATCTGCTTG-3′), *IL-1β* (Forw: 5′-AAAGAAGAAGATGGAAAAGCGATT-3′, Rev: 5′-GGGAACTGGGCAGACTCAAATTC-3′), *IL-6* (Forw: 5′-GGTACATCCTCGACGGCATCT-3′, Rev: 5′-GTGCCTCTTTGCTGCTTTCAC-3′); *CD163* (Forw: 5′-GTCGCTCATCCCGTCAGTCATC-3′, Rev: 5′-GCCGCTGTCTCTGTCTTCGC-3′). Relative quantification of mRNA expression levels was performed according to the ΔCq method, and the expression levels of *36B4* were used as a reference.

### 4.15. Statistics

Results are presented as means ± SE. A one-way ANOVA for repeated measures was used for plasma CK-M and IL-6 level, with Dunnett post-hoc analysis. The mRNA contents were normalized to the geometric mean of *36B4* reference gene, and values were expressed relative to the data obtained at baseline. The level of significance was set at *p* ≤ 0.05. MACSPlex results were analyzed by a one-way ANOVA followed by Tukey’s post-hoc test correction for multiple comparisons.

## 5. Conclusions

Physical exercise is one of the most critical stimuli able to produce significant benefits to the whole organism by affecting cardiovascular, cognitive, and immune functions and energy metabolism. Many papers report that muscle is an endocrine organ that produces and releases myokines, which can influence metabolism and modify cytokine production in tissues and organs. In this inter-organ crosstalk, a new mode of cell communication, mediated by EVs, has emerged. In this study, we highlighted that EV-miRNAs are released in the bloodstream dependent on the fitness condition of the subject; the more the subjects are trained, the less circulating EV-miRNAs increase following an exercise bout. Moreover, although these findings need further investigation, the EV cargo modulation and their biological activity suggest that EVs could have a role in regulating physical adaptations to exercise.

## Data Availability

The data presented in this study are available in this article and supplementary material.

## Supplementary Materials

The following supporting information can be downloaded at: https://www.mdpi.com/article/10.3390/ijms24033039/s1.

## References

[1] Fiuza-Luces C., Santos-Lozano A., Joyner M., Carrera-Bastos P., Picazo O., Zugaza J.L., Izquierdo M., Ruilope L.M., Lucia A. (2018). Exercise Benefits in Cardiovascular Disease: Beyond Attenuation of Traditional Risk Factors. Nat. Rev. Cardiol..

[2] Frazzitta G., Balbi P., Maestri R., Bertotti G., Boveri N., Pezzoli G. (2013). The Beneficial Role of Intensive Exercise on Parkinson Disease Progression. Am. J. Phys. Med. Rehabil..

[3] Goh J., Ladiges W.C. (2014). Exercise Enhances Wound Healing and Prevents Cancer Progression during Aging by Targeting Macrophage Polarity. Mech. Ageing Dev..

[4] Petridou A., Siopi A., Mougios V. (2019). Exercise in the Management of Obesity. Metabolism.

[5] Xie W.-Q., Men C., He M., Li Y., Lv S. (2020). The Effect of MicroRNA-Mediated Exercise on Delaying Sarcopenia in Elderly Individuals. Dose-Response.

[6] Mann S., Beedie C., Jimenez A. (2014). Differential Effects of Aerobic Exercise, Resistance Training and Combined Exercise Modalities on Cholesterol and the Lipid Profile: Review, Synthesis and Recommendations. Sport. Med..

[7] Antunes J.M.M., Ferreira R.M.P., Moreira-Gonçalves D. (2018). Exercise Training as Therapy for Cancer-Induced Cardiac Cachexia. Trends Mol. Med..

[8] Piccirillo R. (2019). Exercise-Induced Myokines with Therapeutic Potential for Muscle Wasting. Front. Physiol..

[9] Pedersen B.K., Febbraio M.A. (2012). Muscles, Exercise and Obesity: Skeletal Muscle as a Secretory Organ. Nat. Rev. Endocrinol..

[10] Bortoluzzi S., Scannapieco P., Cestaro A., Danieli G.A., Schiaffino S. (2005). Computational Reconstruction of the Human Skeletal Muscle Secretome. Proteins Struct. Funct. Bioinform..

[11] Whitham M., Parker B.L., Friedrichsen M., Hingst J.R., Hjorth M., Hughes W.E., Egan C.L., Cron L., Watt K.I., Kuchel R.P. (2018). Extracellular Vesicles Provide a Means for Tissue Crosstalk during Exercise. Cell Metab..

[12] Safdar A., Saleem A., Tarnopolsky M.A. (2016). The Potential of Endurance Exercise-Derived Exosomes to Treat Metabolic Diseases. Nat. Rev. Endocrinol..

[13] Safdar A., Tarnopolsky M.A. (2018). Exosomes as Mediators of the Systemic Adaptations to Endurance Exercise. Cold Spring Harb. Perspect. Med..

[14] Trovato E., di Felice V., Barone R. (2019). Extracellular Vesicles: Delivery Vehicles of Myokines. Front. Physiol..

[15] Frühbeis C., Helmig S., Tug S., Simon P., Krämer-Albers E.-M. (2015). Physical Exercise Induces Rapid Release of Small Extracellular Vesicles into the Circulation. J. Extracell. Vesicles.

[16] Nederveen J.P., Warnier G., Di Carlo A., Nilsson M.I., Tarnopolsky M.A. (2021). Extracellular Vesicles and Exosomes: Insights From Exercise Science. Front. Physiol..

[17] Guescini M., Canonico B., Lucertini F., Maggio S., Annibalini G., Barbieri E., Luchetti F., Papa S., Stocchi V. (2015). Muscle Releases Alpha-Sarcoglycan Positive Extracellular Vesicles Carrying MiRNAs in the Bloodstream. PLoS ONE.

[18] Guescini M., Guidolin D., Vallorani L., Casadei L., Gioacchini A.M., Tibollo P., Battistelli M., Falcieri E., Battistin L., Agnati L.F. (2010). C2C12 Myoblasts Release Micro-Vesicles Containing MtDNA and Proteins Involved in Signal Transduction. Exp. Cell Res..

[19] Forterre A., Jalabert A., Chikh K., Pesenti S., Euthine V., Granjon A., Errazuriz E., Lefai E., Vidal H., Rome S. (2014). Myotube-Derived Exosomal MiRNAs Downregulate Sirtuin1 in Myoblasts during Muscle Cell Differentiation. Cell Cycle.

[20] Annibalini G., Contarelli S., Lucertini F., Guescini M., Maggio S., Ceccaroli P., Gervasi M., Ferri Marini C., Fardetti F., Grassi E. (2019). Muscle and Systemic Molecular Responses to a Single Flywheel Based Iso-Inertial Training Session in Resistance-Trained Men. Front. Physiol..

[21] Lovett J.A.C., Durcan P.J., Myburgh K.H. (2018). Investigation of Circulating Extracellular Vesicle MicroRNA Following Two Consecutive Bouts of Muscle-Damaging Exercise. Front. Physiol..

[22] Estébanez B., Visavadiya N.P., de Paz J.A., Whitehurst M., Cuevas M.J., González-Gallego J., Huang C.-J. (2021). Resistance Training Diminishes the Expression of Exosome CD63 Protein without Modification of Plasma MiR-146a-5p and CfDNA in the Elderly. Nutrients.

[23] Lugli G., Cohen A.M., Bennett D.A., Shah R.C., Fields C.J., Hernandez A.G., Smalheiser N.R. (2015). Plasma Exosomal MiRNAs in Persons with and without Alzheimer Disease: Altered Expression and Prospects for Biomarkers. PLoS ONE.

[24] O’Brien K., Breyne K., Ughetto S., Laurent L.C., Breakefield X.O. (2020). RNA Delivery by Extracellular Vesicles in Mammalian Cells and Its Applications. Nat. Rev. Mol. Cell Biol..

[25] Wahid F., Shehzad A., Khan T., Kim Y.Y. (2010). MicroRNAs: Synthesis, Mechanism, Function, and Recent Clinical Trials. Biochim. Et Biophys. Acta BBA Mol. Cell Res..

[26] Horak M., Novak J., Bienertova-Vasku J. (2016). Muscle-specific microRNAs in skeletal muscle development. Dev Biol..

[27] van Rooij E., Quiat D., Johnson B.A., Sutherland L.B., Qi X., Richardson J.A., Kelm R.J., Olson E.N. (2009). A Family of MicroRNAs Encoded by Myosin Genes Governs Myosin Expression and Muscle Performance. Dev. Cell.

[28] Mccarthy J. (2008). MicroRNA-206: The Skeletal Muscle-Specific MyomiR. Biochim. Biophys. Acta BBA Gene Regul. Mech..

[29] Kim H.K., Lee Y.S., Sivaprasad U., Malhotra A., Dutta A. (2006). Muscle-Specific MicroRNA MiR-206 Promotes Muscle Differentiation. J. Cell Biol..

[30] Singh G.B., Cowan D.B., Wang D.-Z. (2020). Tiny Regulators of Massive Tissue: MicroRNAs in Skeletal Muscle Development, Myopathies, and Cancer Cachexia. Front. Oncol..

[31] Winbanks C.E., Wang B., Beyer C., Koh P., White L., Kantharidis P., Gregorevic P. (2011). TGF-β Regulates MiR-206 and MiR-29 to Control Myogenic Differentiation through Regulation of HDAC4. J. Biol. Chem..

[32] Small E.M., O’Rourke J.R., Moresi V., Sutherland L.B., McAnally J., Gerard R.D., Richardson J.A., Olson E.N. (2010). Regulation of PI3-Kinase/Akt Signaling by Muscle-Enriched MicroRNA-486. Proc. Natl. Acad. Sci. USA.

[33] Liu H.-C., Han D.-S., Hsu C.-C., Wang J.-S. (2021). Circulating MicroRNA-486 and MicroRNA-146a Serve as Potential Biomarkers of Sarcopenia in the Older Adults. BMC Geriatr..

[34] D’Souza R.F., Woodhead J.S.T., Zeng N., Blenkiron C., Merry T.L., Cameron-Smith D., Mitchell C.J. (2018). Circulatory Exosomal MiRNA Following Intense Exercise Is Unrelated to Muscle and Plasma MiRNA Abundances. Am. J. Physiol. Endocrinol. Metab..

[35] Silver J.L., Alexander S.E., Dillon H.T., Lamon S., Wadley G.D. (2020). Extracellular Vesicular MiRNA Expression Is Not a Proxy for Skeletal Muscle MiRNA Expression in Males and Females Following Acute, Moderate Intensity Exercise. Physiol. Rep..

[36] Luchetti F., Canonico B., Arcangeletti M., Guescini M., Cesarini E., Stocchi V., Degli Esposti M., Papa S. (2012). Fas Signalling Promotes Intercellular Communication in T Cells. PLoS ONE.

[37] Asakura A., Seale P., Girgis-Gabardo A., Rudnicki M.A. (2002). Myogenic Specification of Side Population Cells in Skeletal Muscle. J. Cell Biol..

[38] Neuberger E.W.I., Hillen B., Mayr K., Simon P., Krämer-Albers E.-M., Brahmer A. (2021). Kinetics and Topology of DNA Associated with Circulating Extracellular Vesicles Released during Exercise. Genes.

[39] Egan B., Zierath J.R. (2013). Exercise Metabolism and the Molecular Regulation of Skeletal Muscle Adaptation. Cell Metab..

[40] Yáñez-Mó M., Siljander P.R.-M., Andreu Z., Bedina Zavec A., Borràs F.E., Buzas E.I., Buzas K., Casal E., Cappello F., Carvalho J. (2015). Biological Properties of Extracellular Vesicles and Their Physiological Functions. J. Extracell. Vesicles.

[41] Valadi H., Ekström K., Bossios A., Sjöstrand M., Lee J.J., Lötvall J.O. (2007). Exosome-Mediated Transfer of MRNAs and MicroRNAs Is a Novel Mechanism of Genetic Exchange between Cells. Nat. Cell Biol..

[42] Nielsen S., Åkerström T., Rinnov A., Yfanti C., Scheele C., Pedersen B.K., Laye M.J. (2014). The MiRNA Plasma Signature in Response to Acute Aerobic Exercise and Endurance Training. PLoS ONE.

[43] de Gonzalo-Calvo D., Dávalos A., Montero A., García-González Á., Tyshkovska I., González-Medina A., Soares S.M.A., Martínez-Camblor P., Casas-Agustench P., Rabadán M. (2015). Circulating Inflammatory MiRNA Signature in Response to Different Doses of Aerobic Exercise. J. Appl. Physiol..

[44] Cui S.F., Wang C., Yin X., Tian D., Lu Q.J., Zhang C.Y., Chen X., Ma J.Z. (2016). Similar Responses of Circulating MicroRNAs to Acute High-Intensity Interval Exercise and Vigorous-Intensity Continuous Exercise. Front. Physiol..

[45] Gomes C.P.C., Oliveira G.P., Madrid B., Almeida J.A., Franco O.L., Pereira R.W. (2014). Circulating MiR-1, MiR-133a, and MiR-206 Levels Are Increased after a Half-Marathon Run. Biomarkers.

[46] Mooren F.C., Viereck J., Krüger K., Thum T. (2014). Circulating Micrornas as Potential Biomarkers of Aerobic Exercise Capacity. Am. J. Physiol. Heart Circ. Physiol..

[47] Aoi W., Ichikawa H., Mune K., Tanimura Y., Mizushima K., Naito Y., Yoshikawa T. (2013). Muscle-Enriched MicroRNA MiR-486 Decreases in Circulation in Response to Exercise in Young Men. Front. Physiol..

[48] Rome S., Forterre A., Mizgier M.L., Bouzakri K. (2019). Skeletal Muscle-Released Extracellular Vesicles: State of the Art. Front. Physiol..

[49] Kirby T.J., McCarthy J.J. (2013). MicroRNAs in Skeletal Muscle Biology and Exercise Adaptation. Free Radic. Biol. Med..

[50] Miura P., Amirouche A., Clow C., Bélanger G., Jasmin B.J. (2012). Brain-Derived Neurotrophic Factor Expression Is Repressed during Myogenic Differentiation by MiR-206. J. Neurochem..

[51] Williams A.H., Valdez G., Moresi V., Qi X., McAnally J., Elliott J.L., Bassel-Duby R., Sanes J.R., Olson E.N. (2009). MicroRNA-206 Delays ALS Progression and Promotes Regeneration of Neuromuscular Synapses in Mice. Science.

[52] Das U.N. (2019). Circulating Microparticles in Septic Shock and Sepsis-Related Complications. Minerva Anestesiol..

[53] Reich N., Beyer C., Gelse K., Akhmetshina A., Dees C., Zwerina J., Schett G., Distler O., Distler J.H.W. (2011). Microparticles Stimulate Angiogenesis by Inducing ELR+ CXC-Chemokines in Synovial Fibroblasts. J. Cell Mol. Med..

[54] Guescini M., Maggio S., Ceccaroli P., Battistelli M., Annibalini G., Piccoli G., Sestili P., Stocchi V. (2017). Extracellular Vesicles Released by Oxidatively Injured or Intact C2C12 Myotubes Promote Distinct Responses Converging toward Myogenesis. Int. J. Mol. Sci..

[55] Li Y., Yao M., Zhou Q., Cheng Y., Che L., Xu J., Xiao J., Shen Z., Bei Y. (2018). Dynamic Regulation of Circulating MicroRNAs During Acute Exercise and Long-Term Exercise Training in Basketball Athletes. Front. Physiol..

[56] Olivieri F., Prattichizzo F., Giuliani A., Matacchione G., Rippo M.R., Sabbatinelli J., Bonafè M. (2021). MiR-21 and MiR-146a: The MicroRNAs of Inflammaging and Age-Related Diseases. Ageing Res. Rev..

[57] Seiler K.S., Kjerland G.O. (2006). Quantifying Training Intensity Distribution in Elite Endurance Athletes: Is There Evidence for an “Optimal” Distribution?. Scand. J. Med. Sci. Sport..

[58] Tanner R.K., Fuller K.L., Ross M.L.R. (2010). Evaluation of Three Portable Blood Lactate Analysers: Lactate Pro, Lactate Scout and Lactate Plus. Eur. J. Appl. Physiol..

[59] Bentley D.J., Newell J., Bishop D. (2007). Incremental Exercise Test Design and Analysis. Sport. Med..

[60] Newell J., Higgins D., Madden N., Cruickshank J., Einbeck J., McMillan K., McDonald R. (2007). Software for Calculating Blood Lactate Endurance Markers. J. Sport. Sci..

[61] Kenneally M., Casado A., Santos-Concejero J. (2018). The Effect of Periodization and Training Intensity Distribution on Middle- and Long-Distance Running Performance: A Systematic Review. Int. J. Sport. Physiol. Perform..

[62] Théry C., Witwer K.W., Aikawa E., Alcaraz M.J., Anderson J.D., Andriantsitohaina R., Antoniou A., Arab T., Archer F., Atkin-Smith G.K. (2018). Minimal Information for Studies of Extracellular Vesicles 2018 (MISEV2018): A Position Statement of the International Society for Extracellular Vesicles and Update of the MISEV2014 Guidelines. J. Extracell. Vesicles.

[63] van den Berg R.A., Hoefsloot H.C., Westerhuis J.A., Smilde A.K., van der Werf M.J. (2006). Centering, Scaling, and Transformations: Improving the Biological Information Content of Metabolomics Data. BMC Genom..

[64] Prusis P., Lundstedt T., Wikberg J.E.S. (2002). Proteo-Chemometrics Analysis of MSH Peptide Binding to Melanocortin Receptors. Protein Eng. Des. Sel..

[65] Eastment H., Krzanowski W. (1982). Cross-validatory choice of the number of components from a principal component analysis. Technometrics.

[66] Trygg J., Wold S. (2002). Orthogonal projections to latent structures (O-PLS). J. Chemom..

[67] Bro R., Smilde A.K. (2014). Principal component analysis. Anal. Methods.

[68] Adosraku R.K., Choi G.T., Constantinou-Kokotos V., Anderson M.M., Gibbons W.A. (1994). NMR lipid profiles of cells, tissues, and body fluids: Proton NMR analysis of human erythrocyte lipids. J. Lipid Res..

[69] Bonzom P.M., Nicolaou A., Zloh M., Baldeo W., Gibbons W.A. (1999). NMR lipid profile of *Agaricus bisporus*. Phytochemistry.

