# Supplementary material for: Modulation of the Circulating Extracellular Vesicles in Response to Different Exercise Regimens and Study of Their Inflammatory Effects
Source: Int J Mol Sci. 2023 Feb 3;24(3):3039. doi: 10.3390/ijms24033039 (PMC9917742; doi:10.3390/ijms24033039)
Supplement: Supplementary file 1 [file ijms-24-03039-s001.zip › ijms-2168451-supplementary.pdf]

Modulation of the circulating extracellular vesicles in response to different exercise regimens and study of their inflammatory effects

Serena Maggio[1], Barbara Canonico[1], Paola Ceccaroli[1], Emanuela Polidori[1], Andrea Cioccoloni[1], Luca Giacomelli[1], Carlo Ferri Marini[1], Giosuè Annibalini[1], Marco Gervasi[1], Piero Benelli[1], Francesco Fabbri[2], Laura Del Coco[3], Francesco Paolo Fanizzi[3], Anna M. Giudetti[3], Francesco Lucertini[1] and Michele Guescini[1]*

[1] Department of Biomolecular Sciences, University of Urbino Carlo Bo, 61029 Urbino, Italy

[2] Biosciences Laboratory, Istituto Scientifico Romagnolo per lo Studio e la Cura dei Tumori (IRST) IRCCS, Via P. Maroncelli 40, 47014 Meldola, Italy

[3] Dipartimento di Scienze e Tecnologie Biologiche ed Ambientali, Centro Ecotekne, Monteroni,LECCE (LE), Italy

* Correspondence: michele.guescini@uniurb.it

Materials and Methods

Physical activity level

The seven-day physical activity recall questionnaire was administered by a trained interviewer who followed the guidance obtained from Sallis *et al.* for both questionnaire administration and subsequent physical activity level calculations. Subjects were deemed as not physically active – hence enrolled – if both their average daily energy expenditure was lower than 1.8 metabolic equivalents (i.e., higher than the 1.5 value of 'sedentary behaviour' but lower than the 1.8 value of 'standing, talking in person, on the phone, computer, or text messaging, light effort' and if they considered their own last week' physical activity as representative of their habits of the previous 3 months.

Anthropometry and body composition

Weight (barefoot, to nearest 0.5 kg) and height (barefoot and head in the Frankfurt plane, to nearest 0.01 m) were measured and BMI (kg/m2) was calculated for each participant. Body composition was assessed using a bioelectrical impedance analyser (BIA101-ASE model; Akern, Florence, FI, Italy) according to the instructions of the manufacturer.

Pre-exercise heart rate and cardiorespiratory fitness

First, HR was measured for 10 minutes (with the participant standing quietly) using an HR monitor (RS-800 model; Polar, Kempele, Finland). The average HR of the last 5 minutes was assumed as the pre-exercise HR. Then, the participant performed a graded exercise test (GXT) to exhaustion on a treadmill (Runrace model; Technogym, Cesena, FC, Italy) using a personalized incremental running protocol. The GXT started with a warm-up of 5 minutes by walking on the treadmill (set at 1% grade). During the 5th minute, treadmill speed was gradually increased until the participant preferred to start running instead of walking. The preferred walk-to-run transition speed (PTS) was recorded and used as the initial speed of the GXT, which started immediately after the end of the warm-up. Treadmill grade was kept constant at 1% throughout the GXT, while the initial running speed was set at each participant's PTS. The speed increment of each 1-min stage was calculated using the following procedure, which aims to allow the attainment of the VO2peak approximately within 10 minutes from the beginning of

the GXT. First, the maximal $\dot{V}O2$ of the participant was estimated using a simple, gender-specific, non-exercise equation: [height (cm) – age (yrs)] multiplied by 20 (for men) or 14 (for women). The speed yielding the estimated maximal $\dot{V}O2$ (i.e., the postulated final speed of the GXT) was then calculated from the estimated maximal $\dot{V}O2$ according to the ACSM's running metabolic equation, and finally the speed increment of each 1-min stage was calculated as the difference between the final and initial speed divided by 10 (min). Strong verbal encouragement was provided throughout the GXT in order to push the participant to his/her own volitional maximal effort.

For the duration of the test, $\dot{V}O2$ was measured (breath-by-breath) using a metabolimeter (k4b2 model; Cosmed, Rome, Italy) and HR was constantly recorded. Before starting each test, the gas analyzers of the metabolic cart were calibrated using known ambient-air and sample gas references (16% O2, 5% CO2), while the turbine flow meter was calibrated with a syringe of known volume (3 liters). $\dot{V}O2$ and HR raw data were smoothed using the 15-breath moving average and the 5-second stationary time average procedures, respectively. The highest smoothed values of $\dot{V}O_2$ and HR were assumed as $\dot{V}O_{2peak}$ and HRmax, respectively.

Table S1. AT and AMAE participants' characteristics.

	**Age**	**Height**	**Weight**	**BMI**	**HRrest**	**VO_{2max}**
**Mean**	20.1	168	62.3	22	71.1	42.8
**SD (±)**	0.6	8	13.8	3.2	10.6	4.4

All data are reported as mean and standard deviation. Age: years; Height: cm; Weight: kg; BMI: body mass index ($kg \cdot m^{-2}$); FM: fat mass (% of body weight); HRrest: pre-exercise heart rate (beats per minute, bpm); VO_{2max}: maximum VO_2 assessed during the specific test (ml/kg/min).

Table S2. AAT participants' characteristics.

	**Age**	**Height**	**Weight**	**BMI**	**Mileage**	**HR_{max}**	**VO_{2max}**	**FBL2**	**FBL4**
**Mean**	23.3	172.3	61.4	20.6	192.7	194.3	61.8	11.1	13.6
**SD (±)**	6.8	11.4	10	2.8	14.6	8.8	8.6	2.1	2.0

All data are reported as mean and standard deviation. Age: years; Height: cm; Weight: kg; BMI: body mass index ($kg \cdot m^{-2}$); FM: fat mass (% of body weight); Mileage: mileage of the entire training period at a medium altitude (km); HRmax: maximum heart rate measured during the VO2max test (beats per minute, bpm); VO_{2max}: maximum VO_2 assessed during the specific test (ml/kg/min); FBL2: the value of running speed at 2 mmol/l fixed blood lactate (km/h); FBL4: the value of running speed at 4 mmol/l fixed blood lactate (km/h).

NMR analysis

The NMR experiments were recorded on a Bruker Avance III NMR spectrometer (Bruker, Ettlingen, Germany), operating at 600.13 MHz for ^1H observation, equipped with a TCI cryoprobe (Triple Resonance inverse Cryoprobe), incorporating a z-axis gradient coil and automatic tuning-matching. Experiments were acquired at 300 K in automation mode after loading individual samples on a Bruker Automatic Sample Changer, interfaced with the software IconNMR (Bruker). For each sample, a standard 1D ^1H one-dimensional spectrum was acquired (zg Bruker pulse program). The resulting FIDs were multiplied by an exponential weighting function corresponding to a line broadening of 0.3 Hz before Fourier transformation, automated phasing, and baseline correction. The ^1H NMR spectra were processed using Topspin 3.6.1 and Analysis of Mixture Amix 3.9.13 (Bruker, Biospin, Italy) softwares, both for simultaneous visual inspection and the statistical bucketing process. The entire NMR spectra (in the range 10.00-0.5 ppm) were segmented in fixed rectangular buckets of 0.04 ppm width and successively integrated. The spectral region between 7.44-6.28 and 1.80-1.48 ppm

were discarded, excluding the residual peaks of chloroform and water, respectively. The total sum normalization was applied to minimize small differences due to sample concentration and/or experimental conditions among samples. Multivariate statistical analysis (unsupervised principal component analysis (PCA) and the supervised partial least squares discriminant analysis and orthogonal partial least squares discriminant analysis PLS-DA and OPLS-DA) were performed to examine the intrinsic variation in the data, using SIMCA 14 software, (Sartorius Stedim Biotech, Umeå, Sweden) [1-3]. The Pareto scaling procedure was applied, performed by dividing the mean-centered data by the square root of the standard deviation [4].

Results

General overview of lipid extracts

Typical NMR spectra of exosomes lipid extracts with the identified NMR signals are shown in **Figure 1** and related assignments are reported in **Table 1**. 2D COSY, HSQC, HMBC and J-resolved spectra are performed on samples and used to accurately identify and assign the lipid classes present in the samples (saturated, unsaturated fatty acids, phospholipids). The ^{1}H NMR spectrum can be divided into three broad regions: 2.9-0.65 ppm for fatty acids and sterol methyl and methylene resonances (A); 5.1-3.00 ppm for phospholipids head groups and glycerol backbone proton resonances (B); 6.00-5.2 ppm for vinyl protons resonances (C) [5]. Cholesterol is identified by signals in the spectral region between 1.05 and 0.5 ppm. In particular, characteristic singlets of the methyl groups of cholesterol are identified at 1.02 and 0.69 ppm. Choline lipids are identified by the signals at 3.35-3.32 ppm of methyl groups ($N^+(CH_3)_3$). The methylene protons of choline head groups ($-OCH_2CH_2N+$) resonate at 3.65 ppm and is confirmed in the 2D-COSY spectrum, by the cross peaks with 3.94 and 4.17 ppm. All diacylglycerophospholipids (DAGP) are represented by the backbone glycerol sn-2 proton multiplet at 5.23 ppm, together with 4.40 and 4.14 ppm (2D-COSY cross peaks) and 3.98 ppm. Triacylglycerols (TGs) show characteristic signals at 5.27, 4.29-4.27 and 4.15 ppm, partially overlapped with other moieties peaks [5-6]. Signal at 2.38 ppm appears for the presence of n-3 polyunsaturated fatty acids, and other NMR signals at 0.87, 1.30, 1.59 ppm. Finally, broad signals at 5.70-5.65 and 5.48-5.42 ppm show a 2D COSY cross peak, indicating the presence of sphingenine [5].

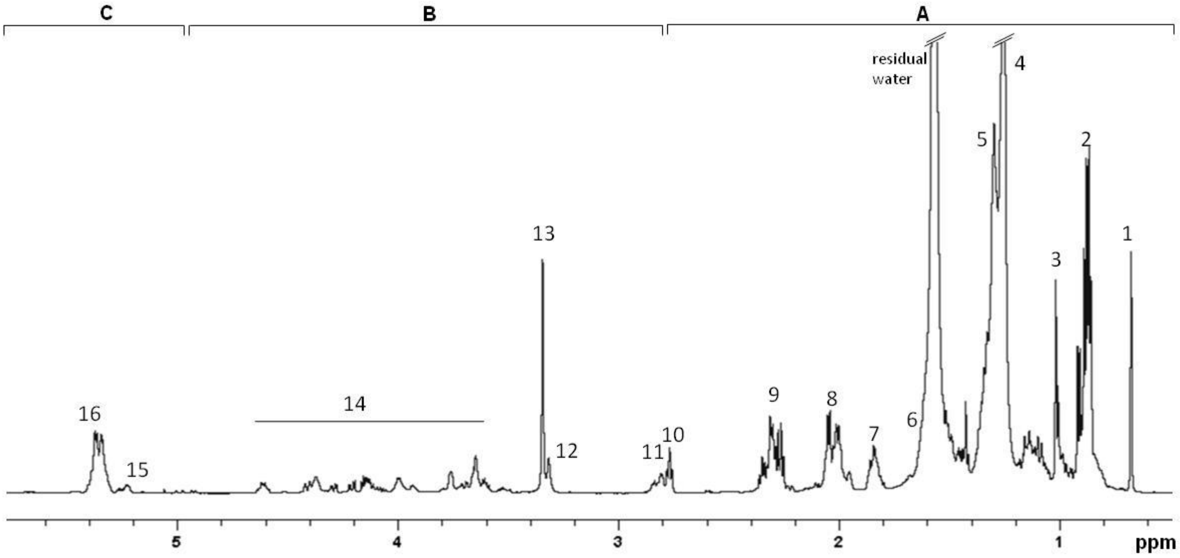

Figure S1. NMR spectra of exosomes lipid extracts with identified NMR signals (region **A**: 2.90-0.65 ppm, typical of fatty acids and sterol methyl and methylene resonances; region **B**: 5.10-3.00 ppm for phospholipids head groups and glycerol backbone proton resonances; region **C**: 6.00-5.20 ppm for vinyl

protons resonances). Numbers are referred to the corresponding signal assignments reported in **Table S3**.

Table S3. Different lipid classes and the corresponding resonance assignments identified by the NMR analysis.

Resonance	^{1}H NMR signal	Chemical shift (ppm)	Lipid class
**1**	–C18H_3 in cholesterol	0.69	Cholesterol
**2**	–CH_3 of all fatty acyl chains	0.89	Total fatty acids
**3**	–C19H_3 in cholesterol	1.02	Cholesterol
**4**	–(CH_2)$_n$– in fatty acyl chain	1.25	Total fatty acids
**5**	=CHCH_2CH$_2$(CH$_2$)– in fatty acyl chain	1.30	Total fatty acids
**6**	–CO-CH$_2$CH_2– in fatty acyl chain	1.62	Total fatty acids
**7**	–CH_2HC= fatty acyl chain UFA	2.02	Unsaturated fatty acids
**8**	–CH_2HC= fatty acyl chain UFA	2.07	Unsaturated fatty acids
**9**	–CO-CH_2– in fatty acyl chain	2.37-2.23	Total fatty acids
**10**	=CHCH_2CH= in fatty acyl chain n-6 PUFA	2.74	Polyunsaturated fatty acids
**11**	=CHCH_2CH= in fatty acyl chain n-3 PUFA	2.88	Polyunsaturated fatty acids
**12**	–N$^+$(CH_3)$_3$ in SM head group	3.29	Sphingomyelins
**13**	–N$^+$(CH_3)$_3$ in PC head group	3.35-3.32	Phosphatidylcholine
**14**	PC and DAGP signals	4.5-3.5	Phosphatidylcholine
**15**	>C2*H* in glycerol backbone of DAGP	5.26	Diacylglycerophospholipids
**16**	–*H*C=C*H*– in fatty acyl chain PUFA and MUFA	5.42-5.29	Polyunsaturated fatty acids

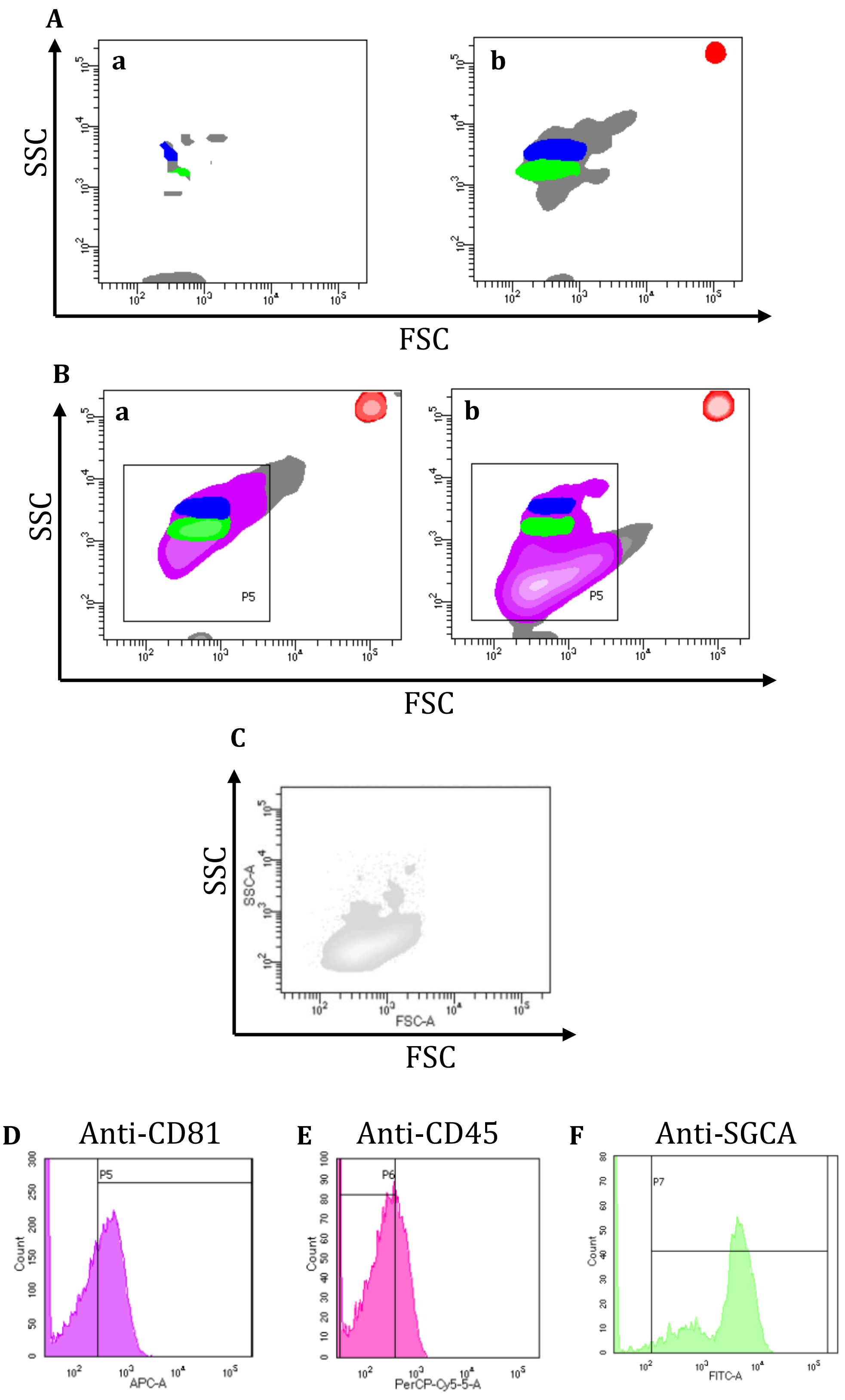

Figure S2. Contour plot FSC vs SSC of EV preparations derived from filtered buffer (**A**a) and from filtered buffer + size beads (**A**b) samples. Green and blue clusters represent the area of beads sized 500 nm and 1 micron, respectively. Such beads are acquired with the goal to establish an area with specific dimensional coordinates, enabling to draw the gates for EV analyses. **B**a and **B**b: Contour plot

FSC vs SSC of EV preparations from Jurkat and from C2C12 cell, respectively. **C**: Contour plot FSC vs SSC of the area obtained after purging unwanted events, in C2C12 EV preparations. The protocol of gating strategy previously applied, is here optimised on C2C12 EV preparation, in order to both validate the sequential gating strategy and to set P7: the gate providing the correct percentage of "true" SGCA positive EVs.

1) Violet events (histogram) are those from the area identified by means of size beads and positive for CD81 (**D**).
2) Pink events (histogram) are those enclosed by P5, drawn in violet histogram, virtually corresponding to appropriate-sized events, positive for CD45 (**E**).
3) Green events (histogram) those enclosed by P6, drawn in pink histogram, virtually corresponding to appropriate-sized events, positive for CD81 and negative for CD45.This histogram focuses SGCA expression (**F**).

References

1. Eastment, H.; Krzanowski, W. Cross-validatory choice of the number of components from a principal component analysis. *Technometrics*. **1982**, *24*, 73-77.

2. Trygg, J.; Wold, S. Orthogonal projections to latent structures (O-PLS). *J. Chemom*. **2002**, *16*, 119-128.

3. Bro, R.; Smilde, A.K. Principal component analysis. *Anal. Methods*. **2014**, *6*, 2812-2831.

4. van den Berg, R.A.; Hoefsloot, H.C.; Westerhuis, J.A.; Smilde, A.K.; van der Werf, M.J. Centering, Scaling, and Transfor-mations: Improving the Biological Information Content of Metabolomics Data. *BMC Genom*. **2006**, *7*, 142. https://doi.org/10.1186/1471-2164-7-142.
5. Adosraku, R. K.; Choi, G. T.; Constantinou-Kokotos, V.; Anderson, M. M.; Gibbons, W. A. NMR lipid profiles of cells, tissues, and body fluids: proton NMR analysis of human erythrocyte lipids. *J. lipid Res*. **1994**, *35*(*11*), 1925-1931.
6. Bonzom, P. M., Nicolaou, A., Zloh, M., Baldeo, W., & Gibbons, W. A. NMR lipid profile of Agaricus bisporus. *Phytochemistry*. **1999**, *50*(*8*), 1311-1321.
